# Facile Preparation of Four-Layer MoS_2_ Nanosheets and Their Application to Organic Light-Emitting Diode

**DOI:** 10.1186/s11671-022-03726-z

**Published:** 2022-09-06

**Authors:** Xingwang Jiang, Jie Cheng, Ping Liu, Qingguo Gao, Liming Liu

**Affiliations:** 1 School of Physics, University of Electronic Science and Technology of China, Chengdu, 610054 China; 2 College of Electron and Information Engineering, University of Electronic Science and Technology of China Zhongshan Institute, Zhongshan, 528402 China

## Abstract

High-quality four-layer molybdenum disulfide (MoS_2_) nanosheets with lateral dimension of about 11 µm were prepared by ultrasonic treatment of MoS_2_ powder with assistance of 1-methyl-2-pyrrolidone (NMP) solvent. The optimal preparation conditions for the preparation of MoS_2_ nanosheets were investigated from the aspects of ultrasonic processing time, ultrasonic power and amount ratio of MoS_2_ powder and NMP solvent. At the same time, the MoS_2_ nanosheets were employed as anode buffer layer in organic light-emitting diode (OLED) with copper nanowire (CuNW) film being anode. MoS_2_ nanosheets can reduce roughness of CuNW film, protect CuNW film from oxidation and improve work function of CuNW film. Experiments show that MoS_2_ nanosheets can significantly improve the current density and brightness of the OLED with CuNW film being anode. The maximum brightness of the OLED with MoS_2_ anode buffer layer is 2.15 times that of the OLED without MoS_2_ anode buffer layer. The current density of the OLED with MoS_2_ anode buffer layer is also obviously increased compared with the OLED without MoS_2_ anode buffer layer.

## Introduction

With the development of two-dimensional layered nanomaterials [1–5], molybdenum disulfide (MoS_2_) as an important class of two-dimensional nanomaterials is widely used in many fields such as lubrication, catalysis, energy storage [6–10], and composite materials. MoS_2_ is a semiconductor material with a special energy band structure. MoS_2_ crystal is an indirect band gap semiconductor material with a band width of about 1.2 eV. The stripped single-layer MoS_2_ nanosheet is a direct band gap semiconductor material, and its band gap width is 1.8 eV, or even 1.9 eV. When MoS_2_ is stripped from bulk materials into MoS_2_ nanosheets, they will show excellent photoelectric properties and have great potential in electroluminescence and other fields. Compared with the zero-energy band gap of graphene, MoS_2_ has a tunable energy band gap, which has a brighter prospect in the field of optoelectronic devices, such as secondary battery [11–13], field effect transistor [14, 15], and gas sensor [16–18]. In addition, in comparison with the commonly-used molybdenum oxide, the absence of dangling bonds in MoS_2_ weakens reacting with other chemical species and accordingly promoting stability [19]. The energy levels of MoS_2_ layer can be further tuned through surface treatment and chemical doping, which facilitates diverse modulation in surface work function. Meanwhile, unlike other buffer layers such as thiophene [20] and CuPC [21, 22], we select a facile method of liquid phase exfoliation which is crucial towards scalable manufacturing with low cost and high throughput production.

Organic light-emitting diode (OLED) has attracted considerable attention as next-generation display because they have the advantages of rich colours, low driving voltage, efficient energy utilization and ultra-thin planar luminescence [23–26]. Traditionally, researchers used Indium Tin Oxides (ITO) as anode to fabricate OLEDs. As one of the most potential substitutes for ITO [27, 28], copper nanowire (CuNW) transparent conductive film (TCF) has become the focus of considerable research activities, because copper has larger reserves and lower price [29]. Compared with silver nanowire (AgNW) film, the above advantages of CuNW film still holds. CuNW TCF has gradually shown its commercial application prospects. When the CuNW film is used as the anode of OLED, its work function is not high, it is easy to be oxidized, and its roughness is relatively high. Therefore, it is necessary to improve oxidation resistance of CuNW TCF, reduce its roughness and improve its work function.

In this paper, MoS_2_ nanosheets were prepared by low-cost ultrasonic treatment for mixture of MoS_2_ powder and 1-methyl-2-pyrrolidone (NMP) liquid. Compared with ultrasonic exfoliation method of MoS_2_ in the past [30, 31], our research on the preparation conditions is more comprehensive. The prepared MoS_2_ nanosheets have four layers and the later dimension is about 11 µm. The MoS_2_ nanosheets were spin-coated on the CuNW TCF to form CuNW/MoS_2_ composite transparent conductive film (CTCF). The performance of the CuNW/MoS_2_ CTCF is better than that of CuNW TCF. MoS_2_ nanosheets has been applied in OLEDs with traditional ITO as anode [32, 33]. Ref. [26] discussed application of MoS_2_ nanosheets in OLEDs with AgNW film as anode. In this paper, we try to research application of MoS_2_ nanosheets in OLEDs with CuNW film as anode. An organic light-emitting diodes (OLED) were prepared with the CuNW film being anode and the MoS_2_ film being anode buffer layer. As the anode buffer layer, the MoS_2_ film can effectively improve oxidation resistance, reduce roughness and improve work function for the CuNW film. The addition of MoS_2_ film can significantly improve the photoelectric properties of the OLED. Experiments showed that the current density and brightness of the OLED with MoS_2_ being anode buffer layer is much better than that of the OLED without MoS_2_ being anode buffer layer.

## Experimental

### Fabrication of Copper Nanowires (CuNWs) and CuNW Film

Copric(II) chloride dihydrate, ascorbic, octadecylamine (ODA) and glacial acetic acid were all purchased from Shanghai Aladdin Biochemical Technology Co. Ltd. In a typical process for synthesizing CuNWs, 95 mg Copric(II) chloride dihydrate, 44 mg ascorbic and 0.7 g ODA were added to 100 ml deionized water in turn. Subsequently, the obtained suspension was transferred into a Teflon-lined autoclave and sealed for 18 h at 130 °C. The reactor was then cooled down to room temperature naturally. The excess chemicals were removed by centrifugal washing with deionized water and glacial acetic acid in turn. The final product was kept in ethanol.

CuNW TCF was prepared on Polyethylene terephthalate (PET) substrates (188 μm thickness). A slight amount of glacial acetic acid solution containing CuNWs was diluted by 500 ml deionized water. By vacuum filtration of CuNWs dispersion at room temperature, CuNW film was formed on a mixed cellulose ester (MCE) filter membrane (0.45 µm aperture). The deposited film was then transferred to the PET substrate by applying uniform pressure, keeping CuNWs facing MCE filter membrane. Finally, the MCE filter membrane was peeled off and the CuNW film was remained on the PET substrate. The thickness of CuNW TCF is about 110 nm.

### Fabrication of MoS_2_ Nanosheets

**Fig. 1:**
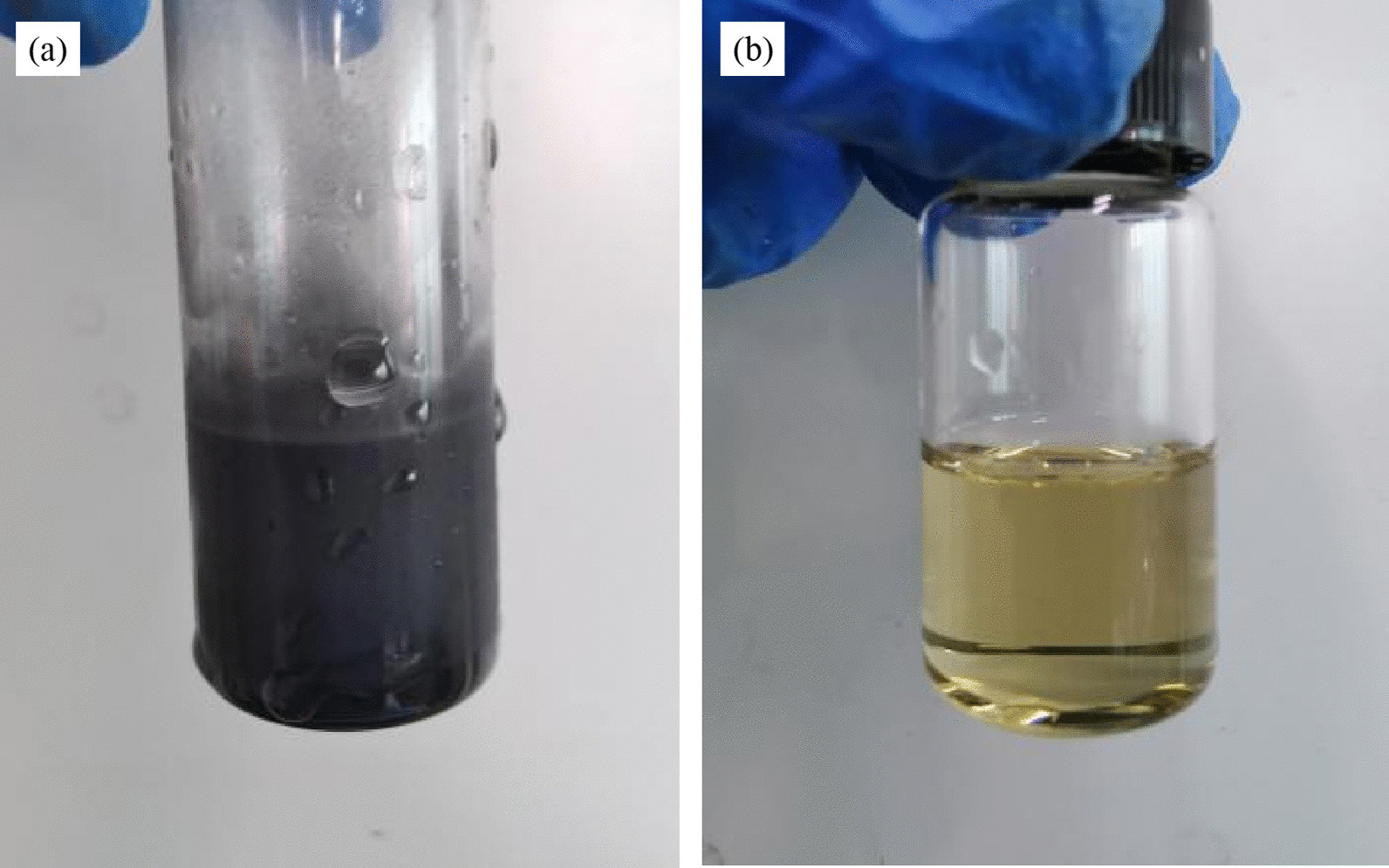
MoS_2_ nanosheet solution. **a** The ultrasonic MoS_2_ solution before centrifugal cleaning, **b** the centrifuged MoS_2_ nanosheet solution

MoS_2_ powder and NMP liquid were both purchased from Shanghai Aladdin Biochemical Technology Co. Ltd. MoS_2_ nanosheets were prepared by ultrasonic exfoliation method. 1 g MoS_2_ powder was added to 25 ml NMP. Then the reagent bottle containing mixture gently placed in an ultrasonic device, which was set to power 360 w, ultrasonic time 12 h and ultrasonic temperature 6 °C. The ultrasonic MoS_2_ solution is black turbid liquid, as shown in Fig. 1a. Then, the ultrasonic solution was centrifuged twice, which can remove MoS_2_ not stripped. The first centrifugal parameter was that the rotating speed was 3500 rpm and the time was 25 min. After the first centrifugal cleaning, we collected supernatant for the second centrifugal cleaning. The second centrifugal parameter was that the rotating speed was 5000 rpm and the centrifugation time was 5 min. The supernatant was collected and stored in the reagent bottle at low temperature. The solution of MoS_2_ nanosheets after centrifugal cleaning is yellow transparent liquid, as shown in Fig. 1b.

### Fabrication of CuNW/MoS_2_ CTCF and CuNW/MoS_2_/PEDOT:PSS CTCF

60 µl MoS_2_ nanosheet solution was coated onto surface of CuNW TCF using a spin coater at 1000 rpm for 10 s and 2000 rpm for 20 s. The thickness of MoS_2_ is about 10 nm. The CuNW/MoS_2_ CTCF was annealed at 90 °C for 1 min.

A drop of dimethyl sulfoxide (DMSO) and a drop of fluorine surfactant (FS-3100) were dripped into a PEDOT:PSS solution (PH 1000) to increase conductivity of PEDOT:PSS, and then the mixed PEDOT:PSS solution was spin-coated at 1000 rpm for 10 s and 2000 rpm for 40 s on the surface of CuNW/MoS_2_ CTCF. The thickness of PEDOT:PSS is about 60 nm. The CuNW/MoS_2_/PEDOT:PSS CTCF was dried at 100 °C for 3 min. Finally, the CuNW/MoS_2_/PEDOT:PSS CTCF was ultraviolet treated for 1 min.

### Fabrication of OLED

**Fig. 2:**
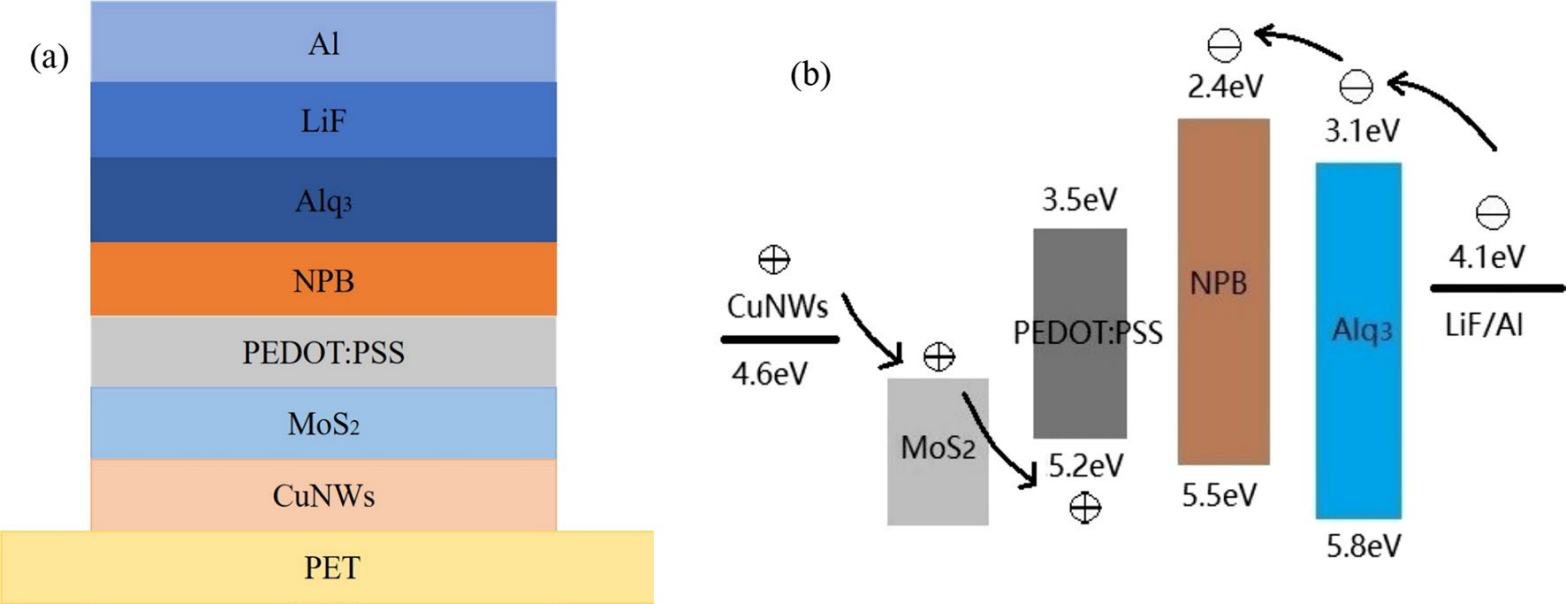
The device structure and energy level of the OLED. **a** The device structure diagram of the OLED. **b** The energy level structure diagram of the OLED

A Green OLED was prepared on the PET substrate coated with CuNW/MoS_2_/PEDOT:PSS CTCF by evaporation in an organic optoelectronic ultra-high vacuum preparation system. N,N′-bis(1-naphthyl)-N,N′-diphenyl-1,1′-biphenyl-4,4′-diamine (NPB) and tris(8-hydroxyquinoline) aluminium (Alq_3_) were received from Sunfine Chemical (South Korea). Lithium fluoride (LiF) and aluminium (Al) were purchased from Kojundo Chemical (Japan). The CuNW/MoS_2_/PEDOT:PSS film was placed on a clean sample tray and then the sample tray was placed into the organic optoelectronic ultra-high vacuum preparation system, followed by vacuum pumping and evaporation of NPB. The evaporation of NPB was carried in an environment where the pressure of the organic chamber was lower than 9.0 × 10^−5^ Pa and the thickness of NPB deposition was 50 nm. Next, Alq_3_ (40 nm), LiF (1 nm) and Al (100 nm) were sequentially deposited onto the sample. The device structure of the OLED is illustrated in Fig. 2a and the corresponding energy level structure is demonstrated in Fig. 2b.

### Characterization

The morphology and dimensions of the prepared MoS_2_ nanosheets were investigated by scanning electron microscope (SEM, JSM-7500F, JEOL), atomic force microscope (AFM, Dimension Edge, BRUKER), Raman spectra (Cora7X00) and X-Ray Diffraction (Bruker, BRUKER OPTICS). The transmittances of CuNW TCF and CuNW/MoS_2_ CTCF were determined by an ultraviolet spectrophotometer (GZ502A). The roughness of CuNW TCF and CuNW/MoS_2_ CTCF was determined by AFM images. The electroluminescent performance of the OLEDs was measured by a Keithley 4200-SCS programmable voltage-current source and ST-86LA screen luminometer.

## Results and Discussion

### Characterization of MoS_2_ Nanosheets

In this work, well-prepared four-layer MoS_2_ nanosheets with lateral dimension of about 11 µm prepared by MoS_2_ powder and NMP solvent. NMP solvent prevented reaggregation of exfoliated nanosheets. Ultrasonic exfoliation method is to mix MoS_2_ powder and NMP solvent according to a certain ratio, some factors such as ultrasonic time, ultrasonic power and content ratio of MoS_2_ powder and NMP solvent have different effects on MoS_2_ nanosheets, and we will discuss these factors in the following subsections.

#### Morphological of MoS_2_ Nanosheets

**Fig. 3:**
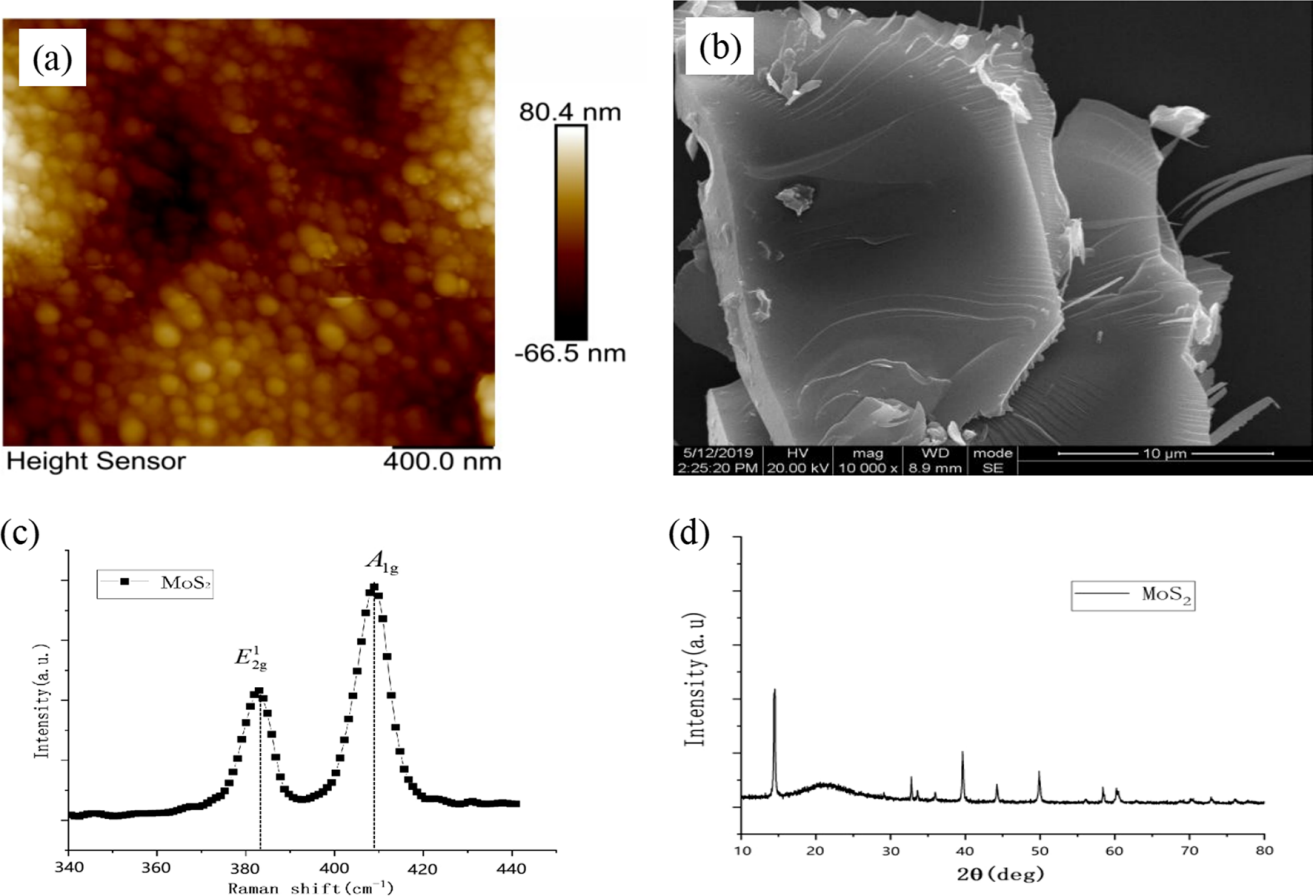
Structure of MoS_2_ nanosheets. **a** AFM image of MoS_2_ nanosheets. **b** SEM image of MoS_2_ nanosheets. **c** Raman spectra of MoS_2_ nanosheets. **d** XRD image of MoS_2_ nanosheets

The AFM image of MoS_2_ nanosheets obtained is shown in Fig. 3a, which characterizes the surface morphology and surface roughness of MoS_2_ nanosheets. It is shown in Fig. 3b that the lateral dimension of MoS_2_ nanosheets is around 11 µm. Figure 3d depicts the X-ray diffraction (XRD) pattern for crystal structure of MoS_2_ nanosheets. It can be seen from the XRD image that there is a strong diffraction peak when 2*θ* = 14.1°, which corresponds to the (002) crystal plane of MoS_2_. And at 2*θ* = 33.1°, a weaker diffraction peak corresponding to (100) plane of MoS_2_ can be found. In order to determine the number of layers of the exfoliated MoS_2_ nanosheets, Raman spectra was used to test the sample, which is shown in Fig. 3c. Two Raman characteristic peaks appeared between 340 and 440 cm^−1^ corresponding to the E^1^_2g_ and A_1g_ vibration modes of MoS_2_, it can be obtained by calculation that Δ = 25 cm^−1^, then we can conclude that the MoS_2_ nanosheets have four layers.

#### Influence of Ultrasonic Time

**Fig. 4:**
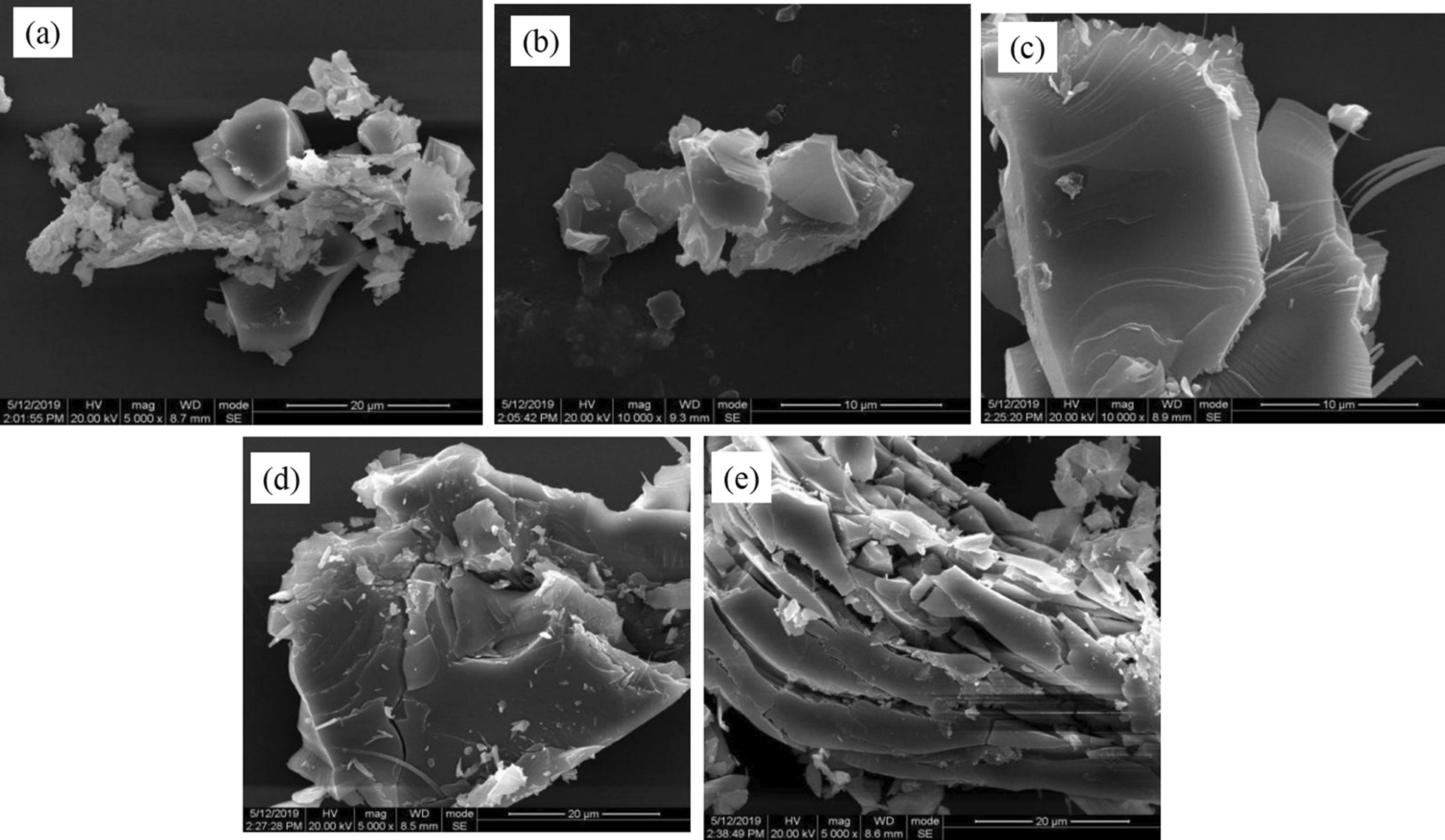
SEM images of MoS_2_ nanosheets under different sonication time. The sonication time are **a** 4 h, **b** 8 h, **c** 12 h, **d** 16 h, **e** 20 h, respectively

In order to observe the preparation process of the MoS_2_ nanosheets, the products were investigated by SEM. Products exhibited different morphologies and size after 4 h to 20 h sonication time. When the sonication time is 4 h, the bulk MoS_2_ is just starting to peel off and there is almost no MoS_2_ nanosheets been produced, which is shown in Fig. 4a. 4 h is too short to break the van der Waals forces between the layers. As shown in Fig. 4b, c, bulk MoS_2_ are exfoliated into nanosheets when time goes from 4 to 12 h. The size of MoS_2_ nanosheets continues to get larger, and the number of nanosheet layers decreases with increasing sonication time. Figure 4d, e indicate that the size of MoS_2_ nanosheets do not become larger within 16 to 20 h. Thus, the suitable sonication time is 12 h.

#### Influence of Ultrasonic Power

**Fig. 5:**
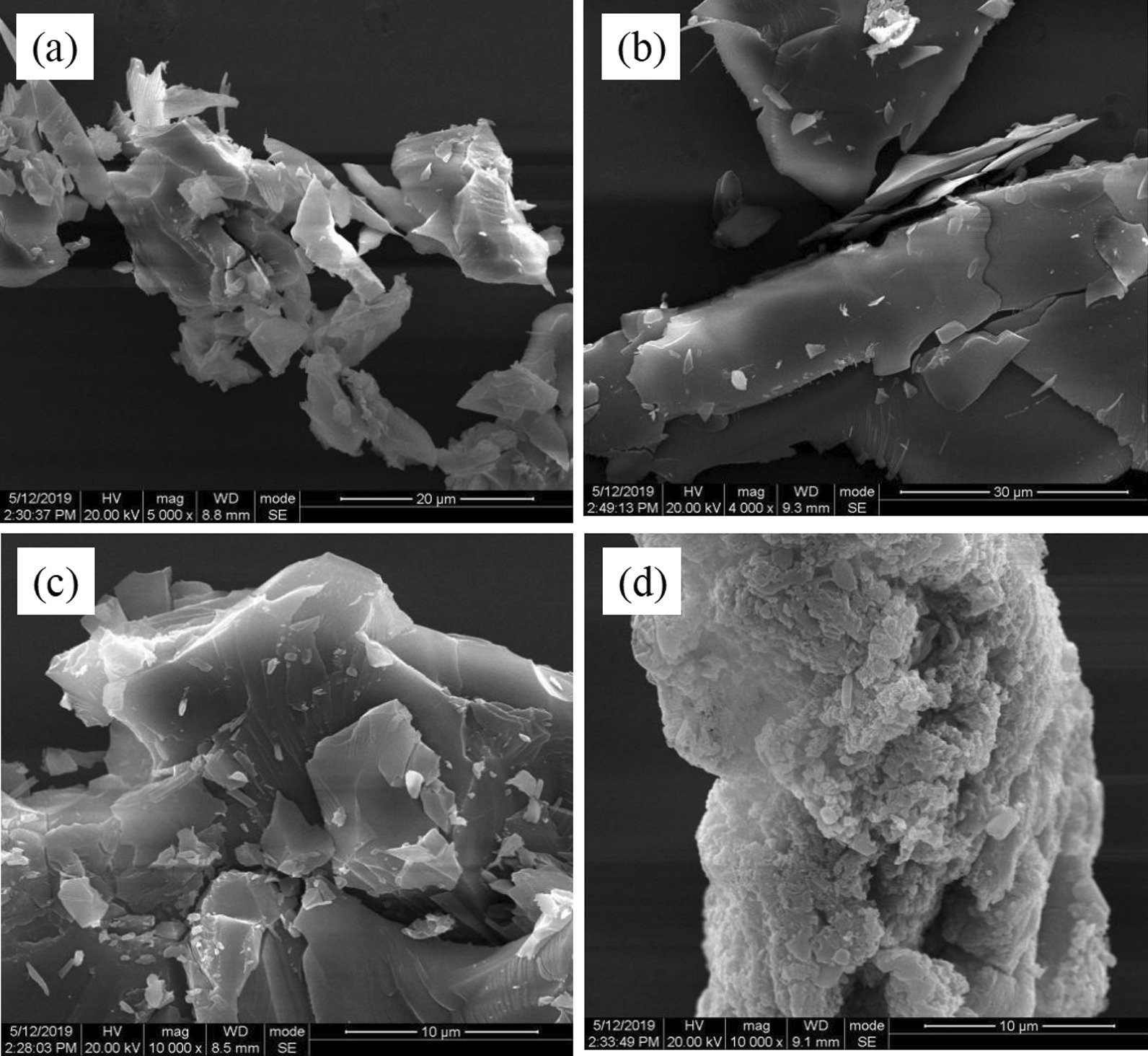
SEM images of MoS_2_ nanosheets under different ultrasonic power. The ultrasonic powers are **a** 240 w, **b** 360 w, **c** 480 w, **d** 600 w, respectively

After two-step centrifugation, the sonicated MoS_2_ nanosheets solution was dropped on the Si sheet and dried naturally for characterization. Different ultrasonic power will lead to different degrees of stripping of MoS_2_ powder, which is shown in Fig. 5. Experimental parameters are demonstrated in Table 1. In Fig. 5a, the products are basically block-shaped and small in size. Few MoS_2_ nanosheets are produced in Fig. 5a, it shows the ultrasonic waves generated by 240 w is not quite enough to interrupt the van der Waals forces between MoS_2_ layers. The bulk material layer-to-layer is completely peeled off when the ultrasonic power is 360 w, as shown in Fig. 5b. When the ultrasonic power is greater than 360 w, the size of the MoS_2_ nanosheets no longer becomes larger. As the ultrasonic power continue to increase, the MoS_2_ nanosheets are broken as shown in Fig. 5c, d. The energy generated by the ultrasound is too large, which cause the covalent bonds in the MoS_2_ nanosheets broken. It can be concluded that the size of MoS_2_ nanosheets is larger when the ultrasonic power is 360 w.

**Table 1:** Exfoliation of MoS_2_ nanosheets with different ultrasonic power

Sample	Time (h)	MoS_2 (_g)	NMP (ml)	Power (w)
A	12	1	25	240
B	12	1	25	360
C	12	1	25	480
D	12	1	25	600

#### Influence of Proportion of MoS_2_ Powder and NMP Solvent

**Fig. 6:**
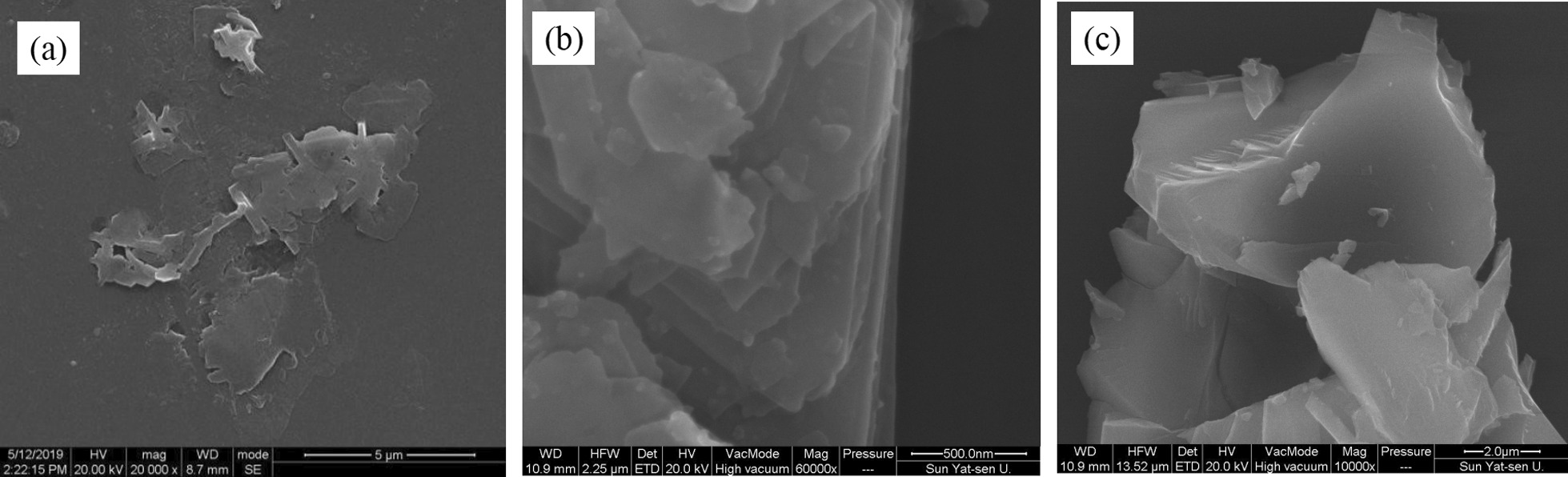
SEM images of MoS_2_ nanosheets prepared with different proportions of MoS2 power and NMP liquid. The volume of NMP is 25 ml, and the masses of MoS_2_ power are **a** 0.5 g, **b** 1.0 g, **c** 1.5 g, respectively

When the ultrasonic time and ultrasonic power were fixed on optimal parameters, different mass ratios of MoS_2_ powder and NMP solvent also lead to different morphologies of MoS_2_ nanosheets. Experimental parameters are shown in Table 2, where NMP solvent has a fixed volume of 25 ml and the quality of MoS_2_ powders varies. As shown in Fig. 6a, the exfoliation of bulk MoS_2_ is not successful due to that the excess NMP solvent is adsorbed on the surface of MoS_2_ through non-covalent bonds. From Fig. 6b, it can be seen that bulk MoS_2_ has been exfoliated into MoS_2_ nanosheets when the mass of MoS_2_ powder is 1 g, and the lateral size is larger than that of Fig. 6a. When the mass of bulk MoS_2_ is 1.5 g, NMP is not enough to weaken the van der Waals force between MoS_2_ layers, so the exfoliated MoS_2_ nanosheets are thicker and multi-layered MoS_2_ nanosheets as shown in Fig. 6c. It indicated that the optimal mass of MoS_2_ powder is 1.0 g when the NMP solvent has a volume of 25 ml.

**Table 2:** The amount ratio of bulk materials and NMP solvent

Sample	Time (h)	Power (w)	MoS_2_ (g)	NMP (ml)
E	12	360	0.5	25
F	12	360	1.0	25
G	12	360	1.5	25

### Optoelectronic Properties of CuNW/MoS_2_ CTCF

**Fig. 7:**
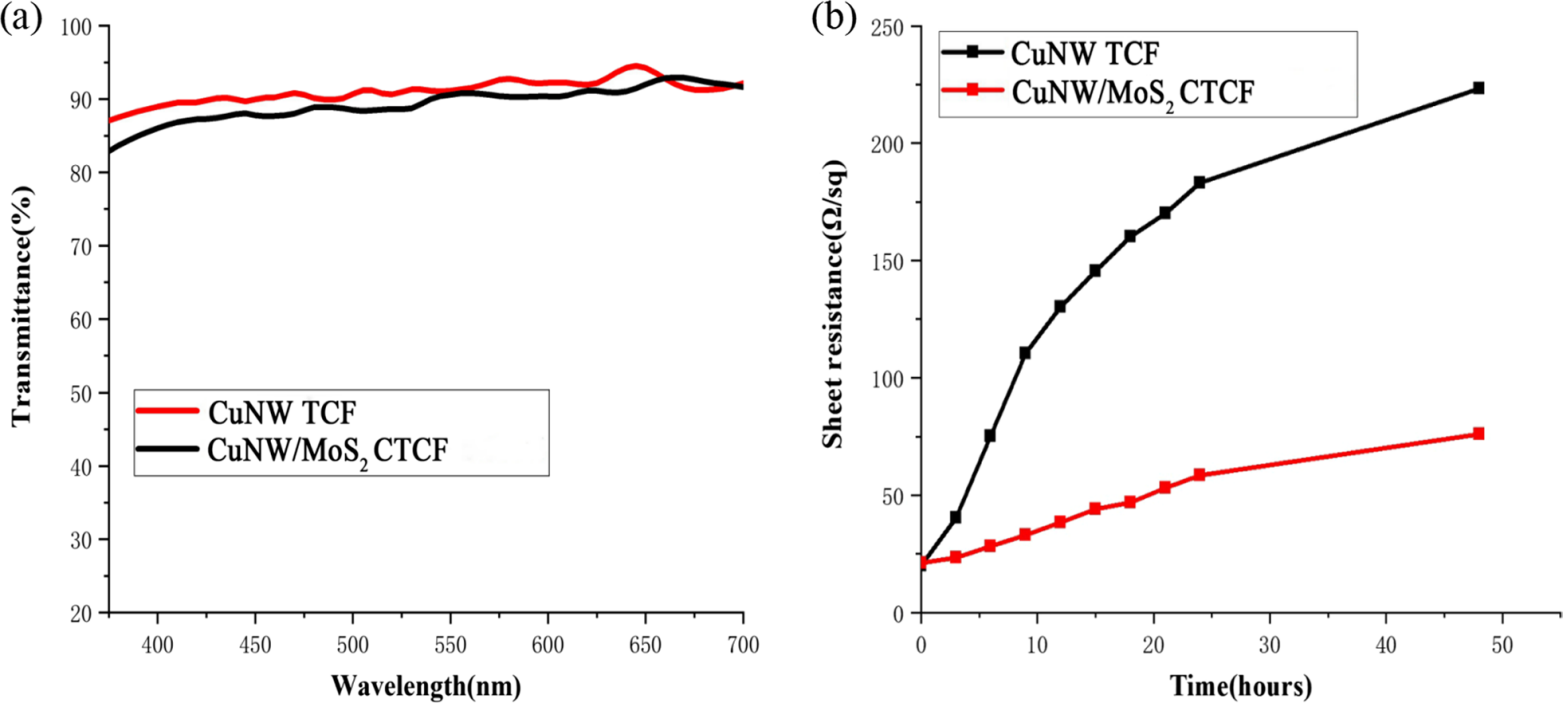
Optoelectronic properties comparison of a CuNW TCF and a CuNW/MoS_2_ CTCF. **a** CuNW TCF and CuNW/MoS_2_ CTCF possess similar transmittance spectra. **b** CuNW TCF and CuNW/MoS_2_ CTCF square resistance curve with time

Although CuNW TCF have many advantages, some fatal shortcomings cannot be ignored, including high roughness, low chemical stability, and low work functions. In order to overcome these problems, CuNW/MoS_2_ CTCF was prepared by spin coating MoS_2_ solution on the surface of CuNW TCF to reduce roughness and increase oxidation resistance and work function of CuNW TCF. The optoelectronic properties of the CuNW TCF and CuNW/MoS_2_ CTCF were compared in Fig. 7. Figure 7a and b show transmittance and sheet resistances of a CuNW TCF and a CuNW/MoS_2_ CTCF on a PET substrate, respectively. Figure 7a shows that the CuNW TCF and the CuNW/MoS_2_ CTCF possess similar transmittance spectra. From Fig. 7b, we can see that sheet resistances of the CuNW TCF and the CuNW/MoS_2_ CTCF were also similar to each other when they have just been prepared. Sheet resistance of the CuNW TCF increased rapidly from 19.92 Ω/sq to 223.25 Ω/sq after 48 h. However, sheet resistance of CuNW/MoS_2_ CTCF showed a slow increase, which increased only 54.74 Ω/sq after 48 h. Thus, the MoS_2_ coating effectively protected the CuNWs from moisture and oxygen.

**Fig. 8:**
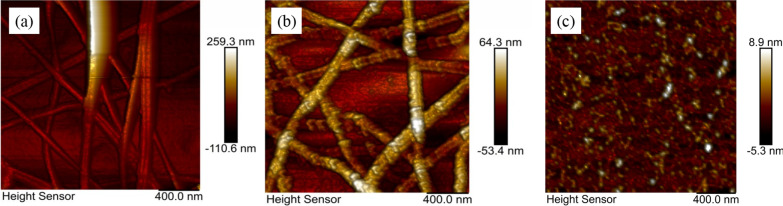
AFM topographic images of **a** CuNW TCF, **b** CuNW/MoS2 CTCF, **c** CuNW/MoS_2_/ PEDOT:PSS CTCF

High roughness will cause short circuits in electronic devices. Therefore, smooth surfaces are crucial to the practical application of the optoelectronic devices. We embedded MoS_2_ into CuNW film to reduce roughness of the CuNW film. Figure 8 demonstrates AFM topographic images of a CuNW TCF and a CuNW/MoS_2_ CTCF. In Fig. 8a, the surface topography of the CuNW TCF is relatively rough. From Fig. 8b, it is obvious that the spin-coated MoS_2_ film can greatly reduce surface roughness of the CuNW film, because the MoS_2_ nanosheets can fill holes between the random grids of CuNWs. In order to further reduce surface roughness of the composite conductive film and improve work function and conductivity of the film, PEDOT:PSS solution was spin-coated on the CuNW/MoS_2_ nanosheet CTCF. Figure 8c shows that the surface roughness of the composite film was reduced a lot.

### Photoelectric Performance of the OLED

**Fig. 9:**
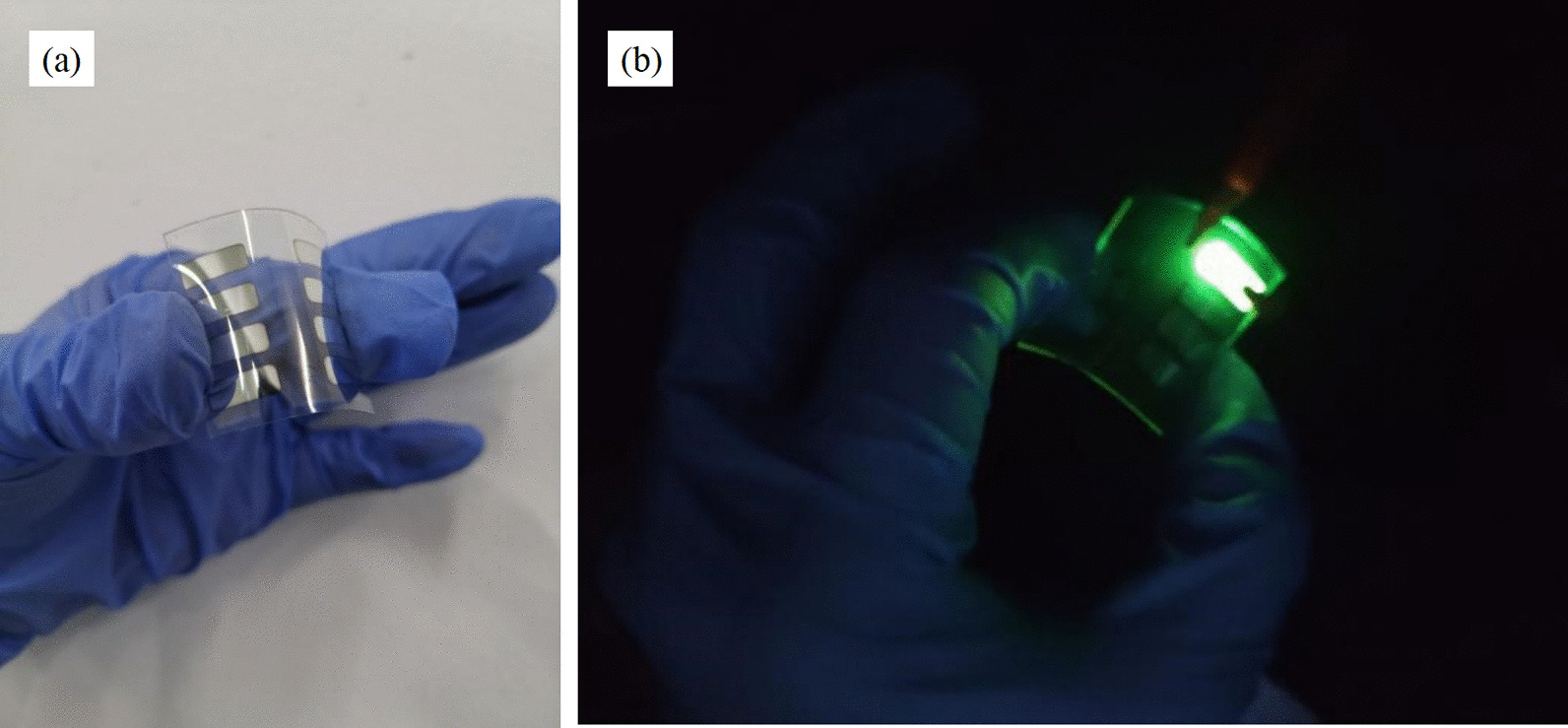
The OLED and light-emitting physical diagram. **a** Diagram of flexible green light OLED, **b** luminescent image of the OLED

We explored the applied of the MoS_2_ nanosheets in flexible OLED. A flexible OLED with CuNW TCF as anode and MoS_2_ nanosheets as anode buffer layer has been prepared. Figure 9a and b demonstrates physical diagram and luminescent image of the flexible OLED, respectively.

**Fig. 10:**
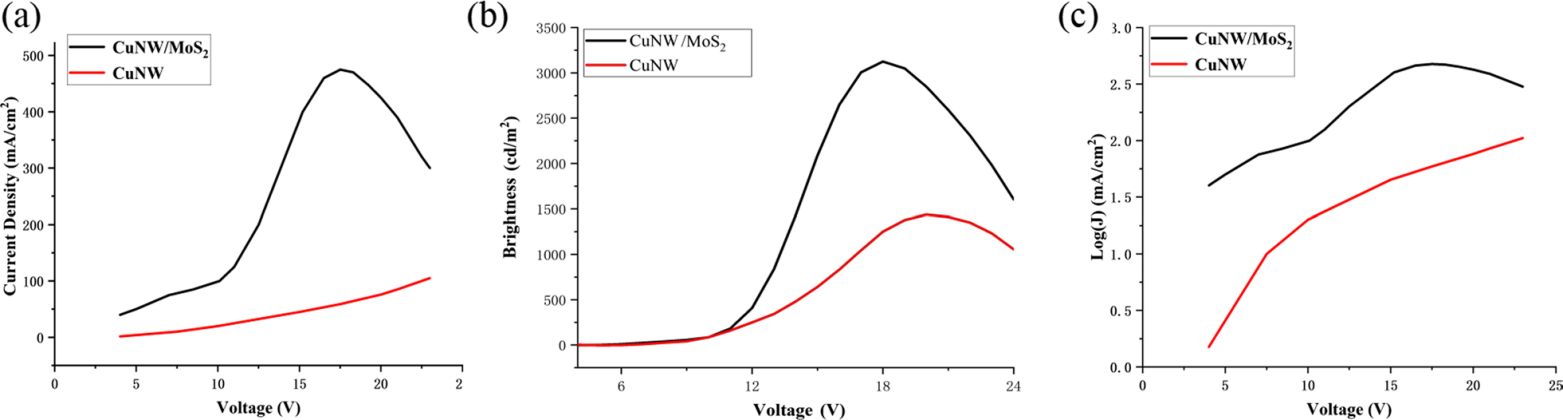
Current density and Brightness of the OLED with MoS_2_ anode buffer layer and the OLED without MoS_2_ anode buffer layer: **a** current density- Voltage characteristics, **b** brightness characteristics, **c** current density in log scale versus voltage

**Fig. 11:**
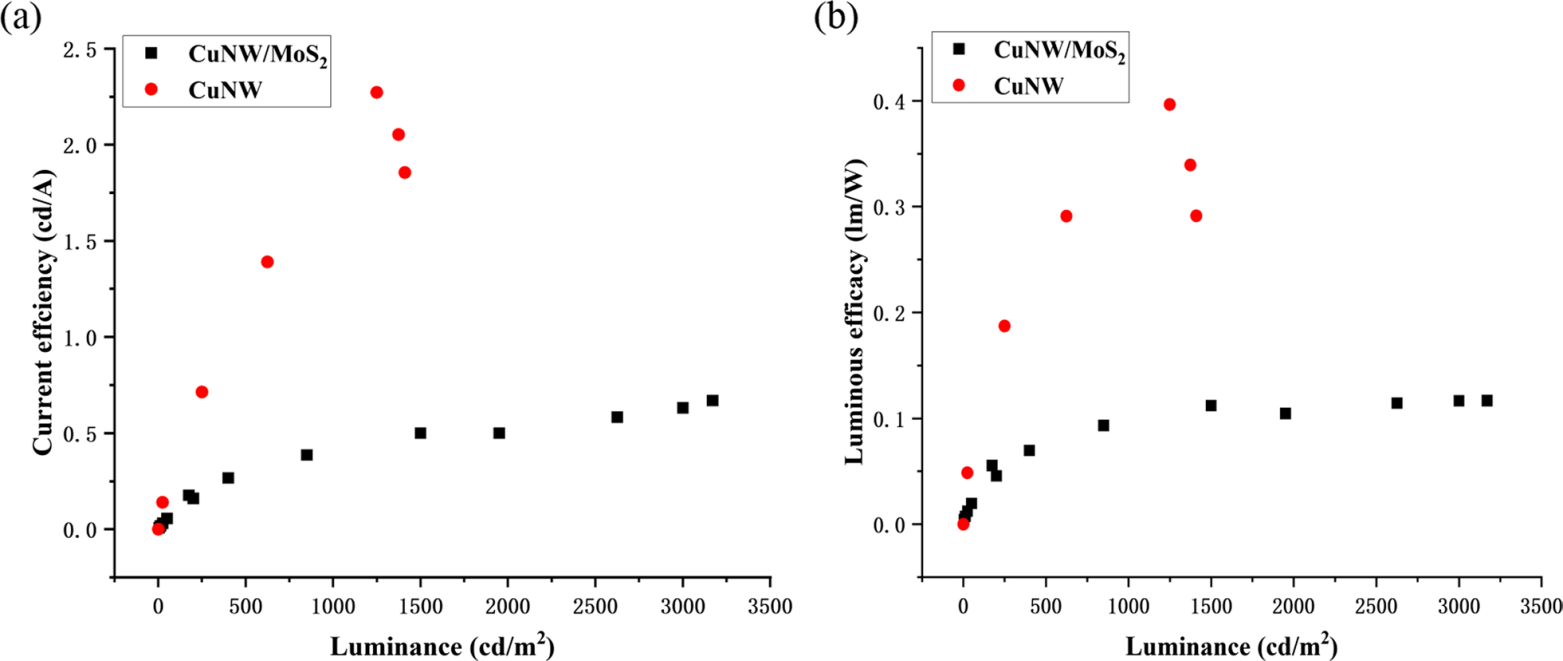
Current efficiency and power efficiency of the OLED with MoS_2_ anode buffer layer and the OLED without MoS_2_ anode buffer layer: **a** current efficiency, **b** power efficiency

All of the measurements were conducted in air at room temperature. The photoelectric properties of the OLEDs with MoS_2_ film and without MoS_2_ film are compared in Fig. 10. At the same driving voltage, current efficiency of the flexible OLED with MoS_2_ anode buffer layer is higher than that of the flexible OLED without MoS_2_ anode buffer layer. As shown in Fig. 10a, the OLED without MoS_2_ anode buffer layer has a maximum current density of 89.34 mA/cm^2^, while the OLED with MoS_2_ anode buffer layer has a maximum current density of 488 mA/cm^2^, which has been enhanced to 5.46 times. MoS_2_ nanosheets have excellent electrical conductivity, which can well modify the surface of CuNWs and improve carrier transport efficiency of the OLED. Figure 10b demonstrates that the OLED with MoS_2_ anode buffer layer has a maximum luminous of 3250 cd/m^2^, while the OLED without MoS_2_ anode buffer layer has only a maximum luminous of 1513 cd/m^2^. The relationship between the two maximum luminous is 2.15 times. Figure 10c is the current density in log scale versus voltage for the OLED with MoS_2_ anode buffer layer and the OLED without MoS_2_ anode buffer layer. At the same voltage, the current density of the MoS_2_-based OLED is greater, indicating that the conductivity of the MoS_2_-based OLED is better. Current efficiency and power efficiency are also important parameters for OLEDs. Figure 11 demonstrates current efficiency and power efficiency of the two type structure of OLEDs. From Fig. 11, we can see that current efficiency and power efficiency of the MoS_2_-based OLED are both lower than that of the OLED without MoS_2_ anode buffer layer. When high energy efficiency is required, the role of MoS_2_ in this device structure needs to be further explored.

## Conclusions

Four-layer MoS_2_ nanosheets with lateral dimension of about 11 µm are prepared by ultrasonic exfoliation method in this paper. The experimental supplies are bulk material MoS_2_ and NMP solvent. Fabrication optimal parameters of the high-quality MoS_2_ nanosheets are ultrasonic processing time for 12 h, ultrasonic power for 360 w, MoS_2_ powder for 1.0 g and NMP solvent for 25 ml. In this paper, a flexible OLED with CuNW film as anode and MoS_2_ as anode buffer layer was fabricated. Compared with the flexible OLED without MoS_2_ anode buffer layer, the current density of the OLED with MoS_2_ anode buffer layer was increased to 5.46 times and the maximum brightness of the OLED was increased to 2.15. MoS_2_ layer can improve the work function of CuNW film, which makes hole injection easier. Furthermore, MoS_2_ nanosheets with large size could fully cover the CuNW film surface, thus, lowering the roughness and oxidation of the CuNW film.

## Data Availability

The datasets supporting the conclusions of this article are included within the article.

## Abbreviations

MoS_2_: Molybdenum disulfide
OLED: Organic light-emitting diode
CuNW: Copper nanowire
ITO: Indium Tin Oxides
TCF: Transparent conductive film
NMP: 1-Methyl-2-pyrrolidone
CTCF: Composite transparent conductive film
CuNWs: Copper nanowires
MCE: Mixed cellulose ester
ODA: Octadecylamine
PET: Polyethylene terephthalate
DMSO: Dimethyl sulfoxide
NPB: N,N′-bis(1-naphthyl)-N,N′-diphenyl-1,1′-biphenyl-4,4′-diamine
Alq_3_: Tris(8-hydroxyquinoline) aluminium
LiF: Lithium fluoride
Al: Aluminium
SEM: Scanning electron microscope
AFM: Atomic force microscope
XRD: X-ray diffraction
